# Standardized nursing and clinical efficacy of OxyContin in reducing oral mucosal pain in patients with nasopharyngeal carcinoma: A randomized, double-blind, placebo-controlled study protocol

**DOI:** 10.1097/MD.0000000000023205

**Published:** 2020-12-04

**Authors:** Nina Sun, Yunxia Li, Peihua Nie

**Affiliations:** a Department of otolaryngology; b Cancer Centre, Shandong Provincial Third Hospital, Shandong, China.

**Keywords:** nasopharyngeal carcinoma, oral mucosal, OxyContin, protocol, standardized nursing

## Abstract

**Background:**

Pain caused by oral mucositis (OM) is the main problem in the process of concurrent chemoradiotherapy for the nasopharyngeal carcinoma (NPC). This protocol aims to explore the standardized nursing and therapeutic effect of OxyContin on OM pain in the patients with NPC undergoing the concurrent chemoradiotherapy.

**Methods:**

The experiment is a randomized clinical research, which was granted through the Research Ethics Committee of Shandong Provincial Third Hospital (No.20200802097). In this research, 90 NPC patients with OM induced by chemotherapy are enrolled, and the score of visual analogue >5 and the grade of OM >1 are evaluated. Patients with known allergy to OxyContin, the opioid abuse history, or major organ dysfunction, for instance, hepatic insufficiency, renal failure, and respiratory and heart failure, as well as a series of severe mental illness are excluded from our research. Patients in study groups receive standardized nursing and oral OxyContin. Patients in control groups only receive oral OxyContin. The analgesic effect could be assessed with the comparison of the visual analogue scale after and before the treatment. Safety evaluations contain the assessment of the vital signs, laboratory tests, as well as adverse events. The Karnofsky performance status standards of the International Cancer Control Union is utilized to evaluate the quality of life.

**Results:**

The comparison of outcomes after taking OxyContin in both groups will be shown in Table 1

**Table 1:** The comparison of outcomes after taking OxyContin in both groups.

Variables	Study group (n = 45)	Control group (n = 45)	*P* value
Pain relief			
Quality of life measures before and after treatment			
Psychological field			
OxyContin consumption			
Complications			

.

**Conclusion:**

The combination of OxyContin and standardized nursing care appears to improve the analgesic efficacy and life quality in NPC patients.

Trial registration: We registered this protocol in Research Registry (researchregistry6098).

## 1 Introduction

Nasopharyngeal carcinoma (NPC) is a kind of epithelial carcinoma, which occurs in the lining of nasopharyngeal mucosa.^[1,2]^ In the nasopharynx, tumors generally occur in pharyngeal recess.^[3]^ Although the NPC originates from similar tissue or cell lineages, it is significantly different from head and neck epithelial neoplasms. Compared with other cancers, NPC is relatively rare. On the basis of the statistics of the International Agency for Research on Cancer, there were approximately 129,000 new NPC cases in 2018, which accounts for only 0.7% of the total number of confirmed cancers in 2018.^[4]^ According to reports, about 70% of cases were diagnosed as stage III and stage IV owing to the biological behavior of NPC.

In the last 20 years, the clinical trials have demonstrated that the concurrent chemorotherapy is beneficial to improve overall survival and disease-free survival of locally advanced NPC patients, and it has become a standard treatment to treat the patients with locally advanced NPC.^[5,6]^ Nevertheless, the concurrent chemoradiotherapy is related to the increase of toxicity, and the oral mucositis (OM) is familiar.^[7,8]^ Wee et al^[9]^ have reported that in comparison with radiotherapy alone, the concurrent chemoradiotherapy led to an obvious increase in the number of patients with grade 3–5 OM. OM often accompanied by pain, this behavior affects the patients life quality, at the same time, it will disrupt the patients normal physiological functions. At present, there are few approaches to alleviate and prevent the ON pain results from the concurrent chemoradiotherapy. There are currently few strategies for preventing and reducing OM pain caused by concurrent chemoradiotherapy, and the primary treatments are oral cleaning, promotion of local mucosal recovery, nutritional supplements, antibiotics, and analgesics.^[7,10]^ Published studies have reported that standardized pain care ensures analgesia, safety and effectiveness of medication, timely detection, and treatment of adverse reactions. Few studies focused on the combination OxyContin with standardized nursing to treat OM. This protocol aims to explore the standardized nursing and therapeutic effect of OxyContin on OM pain in NPC patients with concurrent chemoradiotherapy.

## 2 Materials and methods

The experiment will be implemented from December 2020 to December 2021 at Shandong Provincial Third Hospital, in accordance with the purposes of the Declaration of Helsinki. The experiment was granted through the Research Ethics Committee of Shandong Provincial Third Hospital (No.20200802097) and recorded in research registry (researchregistry6098). The experiment is a single-center randomized clinical research. The recruited patients are given the written informed consent before registration.

### 2.1 Inclusion and exclusion criteria

90 NPC patients with OM induced by chemotherapy are enrolled, and the score of visual analogue >5 and the grade of OM >1 are evaluated. Patients with known allergy to OxyContin, the opioid abuse history, or major organ dysfunction, for instance, hepatic insufficiency, renal failure, and respiratory and heart failure, as well as a series of severe mental illness are excluded from our research.

### 2.2 Nursing method

Patients in study groups (n = 45) receive standardized cancer pain nursing, including: pain assessment based on visual analogue scale. The nurses evaluate the pain of patients 4 times within 24 hour. Following pain control, the status is evaluated every 24 hour. The pain score scale, number of radiotherapy sessions, and OxyContin doses are recorded. Establish a pain care sheet and record the pain characteristics, location, time, medication, and nursing measures: The nurses will determine the pain score based on the highest pain of the patients within 24 hour. Routine care is given for the OM, including antibacterial mouthwash containing bismuth and Vitamin B12. The patient maintains a pain diary. This is combined with illustrations to circle the pain location, record the pain score, describe the pain characteristic with the provided words, and write down the name and dosage of the painkiller and any side effects. It is convenient for the doctors and nurses to check the patients pain, and for the patients to understand their own pain problems. Pain education for patients: Our nurses distribute educative materials such as “Pain Patient Education Handbook” to NPC patients, and explain the drug-related adverse reactions to correctly understand the pain and be able to treat side effects actively. One-on-one teaching is conducted based on the different educational backgrounds of patients, videos are played about cancer pain, and pain-related lectures conducted once a month to increase compliance and improve satisfaction among patients. Psychological care is given to help patients relieve their tension and anxiety caused by pain. Patients in control groups (n = 45) only receive oral OxyContin for pain management.

### 2.3 Observation index

The analgesic effect could be assessed with the comparison of the visual analogue scale after and before the treatment. Safety evaluations contain the assessment of the vital signs, laboratory tests, as well as adverse events. The Karnofsky performance status standards of the International Cancer Control Union is utilized to evaluate the quality of life. We calculate the total dose of OxyContin taken per patient, from the 15th radiotherapy session and the doses have reached 30 to 35 gray at the end of radiotherapy.

### 2.4 Statistical analysis

All the data could analyzed through IBM SPSS Statistical software Windows version 20 (IBM Corp., Armonk, NY, USA). Afterwards, all the data are described with appropriate characteristics such as mean, median, standard deviation as well as percentage. Continuous and categorical variables are analyzed using the independent *t* tests and χ^2^-tests. For the significance level, the value of *P* needed to be less than .05.

## 3 Results

The comparison of outcomes after taking OxyContin in both groups will be shown in Table 1.

## 4 Discussion

NPC is deemed sensitive to radiation.^[11]^ In recent times, radiotherapy has been recognized as a radical treatment. Radiotherapy alone is commonly used in the early stage, and radiotherapy-based radiotherapy and chemotherapy are the main methods employed in the advanced stage. Radiotherapy could effectively improve the prognosis of patients, but adverse reactions such as dry mouth, nausea, vomiting, sore throat, oral ulceration, and skin pigmentation of the neck, occurred easily during treatment.^[12,13]^ Radioactive OM usually appears around 1 to 2 weeks following radiation therapy, often accompanied by changes in taste, dry mouth, saliva, and pain.^[14,15]^ Currently, there is no good approaches for the management of OM pain. The generally utilized analgesics are the drugs of local anesthetics. OxyContin has a good analgesic effect on patients with NPC. The analgesic effect of OxyContin is rapid, long-lasting, stable and has a good tolerance in the patients with distinct degrees of pain.^[16]^ For the moderate pain patients, OxyContin could effectively improve the quality of life; however, for patients with severe pain, it do not significantly improve the quality of life, which could be related to the overall impact of tumor-induced pain in patients. In addition, OxyContin could alleviate the pain in patients but caused some adverse reactions, such as vomiting, constipation, dizziness, and dysuria. https://www.cancerjournal.net/article.asp?issn=0973–1482;year=2018;volume=14;issue=7;spage=1594;epage=1599;aulast=Hu-ref14 The standardized pain care can effectively improve these symptoms.

## 5 Conclusion

The combination of OxyContin and standardized nursing care appears to improve the analgesic efficacy and quality of life in patients with NPC.

## Author contributions

Nina Sun writes the manuscript; Yunxia Li collects and analyzes data; Peihua Nie designs the manuscript. All author approve the submission.

**Data curation:** Yunxia Li.

**Formal analysis:** Yunxia Li.

**Funding acquisition:** Peihua Nie.

**Investigation:** Yunxia Li.

**Writing – original draft:** Nina Sun.

**Writing – review & editing:** Peihua Nie.
